# Shock Following Extensive Muscle Injury

**Published:** 1918-10

**Authors:** 


### SHOCK FOLLOWING EXTENSIVE MUSCLE INJURY

Major W. B. Cannon, Chairman of the Committee on Shock of the Research Committee of the American Red Cross in France, at the September meeting, made a report of experiments on shock in relation to muscle injury that were begun by him in co-operation with Professor Bayliss in London, and reported to the Research Society on March 15 (see *Medical Bulletin*, April, 1918, p. 426). These experiments have been continued recently at the Laboratory of Surgical Research of the American Expeditionary Forces.

Major Cannon said that "evidence has been obtained in support of the following statements :

1. The crushing of muscles in a hind leg is followed by a fall of arterial pressure beginning in about 20 minutes and reaching in about an hour a shock level.

2. This effect occurs even though nerves to the leg are severed ; it is therefore not of nervous origin.

3. If the blood vessels of the leg are tied and the muscle injured, the pressure drops only after the blood flow is restored.

4. If the shock pressure has been caused by muscle injury, tying the vessels may be followed by a progressive rise of pressure to the normal level.

5. The effects of muscle injury may pass away with spontaneous recovery of pressure.

6. The lowered pressure after injury is not primarily due to extravasation of blood and lymph into the injured tissues. The measured amount is insufficient ."

General Bowlby, Director of Surgery of the British Armies in France, who was present at the meeting of the Research Committee which, by the way, always meets immediately following the afternoon session of the Research Society, said that these observations had a practical bearing in surgery, since they proved that the longer a completely smashed limb is left without amputation, the worse the shock. It is, therefore, important that the completely crushed limb be removed as early as possible; and that, if shock is due to absorption of materials from damaged tissues, after an extensive muscle wound a tourniquet applied as tightly as possible after the injury would reduce shock. General Bowlby also said that he had observed clinically that cutting across completely smashed tissues helps the patient.

Major Cannon said that the conclusion to be drawn from these observations are the necessity of keeping the blood pressure of shock patients from falling below a critical level of 80 mm. of mercury, and the need for the surgeons to be prepared to operate as soon as possible after injuries in which there is great damage to muscular tissue.

